# 

**Published:** 1876-02

**Authors:** 


### Books and Pamphlets Received.

Medical Diagnosis, with Special Reference to Practice of Medicine. A Guide to the knowledge and discrimination of Disease. By J. M. DaCosta, M. D. Fourth edition. Philadelphia: J. B. Lippincott & Co., 1876. Buffalo, T. Butler & Son.

A Treatise on the Diseases of Infancy and Childhood. By J. Lewis Smith, M. D. Third edition. Philadelphia: Henry C. Lea, 1876. Buffalo: T. Butler & Son.

Extra-Uterine Pregnancy: its Causes, Species, Pathological Anatomy, Clinical History, Diagnosis, Prognosis and Treatment. By John S. Parry, M. D. Philadelphia: Henry C. Lea, 1876. Buffalo: T. Butler & Son.

The Medical Jurisprudence of Insanity. By J. H. Balfour Brown, Esq. Second edition with reference to Scotch and American decisions. Philadelphia: Lindsay & Blakiston, 1876. Buffalo: T. Butler & Son.

The Body and its Ailments: A Handbook of Familiar Directions. By George H. Napheys, A. M., M. D. Illustrated. Philadelphia: H. C. Watts & Co., 1876.

Archives of Ophthalmology and Otology. Vol. V., No. 1. New York: Wm. Wood & Co., 1876.

Transactions of the American Opthalmological Society. Eleventh Annual Meeting. New York: Wm. Wood & Co., 1876.

The Practitioner. Edited by Lauder Brunton, M. D., F. R. S. Macmillan & Co., London & New York.

Statistics of the Births, Marriages and Deaths in the City of Philadelphia for the year 1874.

Transactions of the Colorado Territorial Medical Society at its Third and Fourth Annual Sessions, Denver, June, 1874–5.

Remarks on Intra-Uterine Polypi, with Special Reference to their Treatment. By A. Reeves Jackson, A. M., M. D. Reprint from the Chicago Medical Journal and Examiner.

The treatment of the Scrofulides (Lupus). By Henry G. Piffard, A. M., M. D. Reprint from the London Practitioner.

Transactions of the Twenty-Fifth Aniversary Meeting of the Illinois State Medical Society, May, 1875.

A Series of American Clinical Lectures. Edited by E. C. Seguin, M. D. Vol. I., No. XI. On the Diagnosis of Diseases accompanied with real or apparent Paraplegia without Muscular Degeneration. By H. C. Wood, Jr., M. D. Vol. I., No. XII. On the Nature of the Gouty Vice. By W. H. Draper, M. D. New York: G. P. Putnam's Sons.

Electro Therapeutics. By Z. C. McElory, M. D. Reprint from the Cincinnati *Lancet and Observer*, January, 1876.

### Contents of February Number.

### ORIGINAL.

**THE BUFFALO**

### Medical and Surgical Journal

**PUBLISHED MONTHLY,**

Containing Original Articles, Reports of Medical Societies and Hospitals, Editorial, Reviews, Correspondence, Army News, etc., etc.; including the usual variety of Medical Periodical Publications. Specimen copies sent on application.

Address Buffalo Medical & Surgical Journal, Buffalo, N. Y.

TERMS OF SUBSCRIPTION, $3.00 PER YEAR, IN ADVANCE.

